# Predator-Prey Interactions Shape Thermal Patch Use in a Newt Larvae-Dragonfly Nymph Model

**DOI:** 10.1371/journal.pone.0065079

**Published:** 2013-06-03

**Authors:** Lumír Gvoždík, Eva Černická, Raoul Van Damme

**Affiliations:** 1 Institute of Vertebrate Biology AS CR, Brno, Czech Republic; 2 Department of Biology, University of Antwerp, Wilrijk, Belgium; University of Sydney, Australia

## Abstract

Thermal quality and predation risk are considered important factors influencing habitat patch use in ectothermic prey. However, how the predator’s food requirement and the prey’s necessity to avoid predation interact with their respective thermoregulatory strategies remains poorly understood. The recently developed ‘thermal game model’ predicts that in the face of imminent predation, prey should divide their time equally among a range of thermal patches. In contrast, predators should concentrate their hunting activities towards warmer patches. In this study, we test these predictions in a laboratory setup and an artificial environment that mimics more natural conditions. In both cases, we scored thermal patch use of newt larvae (prey) and free-ranging dragonfly nymphs (predators). Similar effects were seen in both settings. The newt larvae spent less time in the warm patch if dragonfly nymphs were present. The patch use of the dragonfly nymphs did not change as a function of prey availability, even when the nymphs were starved prior to the experiment. Our behavioral observations partially corroborate predictions of the thermal game model. In line with asymmetric fitness pay-offs in predator-prey interactions (the ‘life-dinner’ principle), the prey’s thermal strategy is more sensitive to the presence of predators than vice versa.

## Introduction

Due to the all-pervading effects of temperature on physiological performance, many organisms have evolved thermoregulatory mechanisms to avoid suboptimal body temperatures. Among mobile ectotherms, the choice of thermally favorable habitat patches is an important and widespread way of maximizing fitness by reducing body temperature variation in a heterogeneous environment [1]–[3]. However, although important, temperature is obviously not the only environmental factor an animal moving through the habitat has to take into account. Local variations in food availability, competition, mating opportunities and predation risk may also influence patch quality [4]–[8]. Recognizing this fact, theoretical models of behavioral thermoregulation have considered both the benefits and the costs of maintaining a particular body temperature [9], [10], [3]. Measurable costs of thermoregulation include energy expenses [9], time consumption [11], and predation risk [10].

The influence of predation risk on prey thermoregulatory behavior or microhabitat choice has received some attention from empiricists [12]–[14]. However, such studies typically consider predators ‘an abstract source of risk to prey rather than participants of predator-prey interaction’ [15]. They often describe behavioral responses of prey to some ubiquitous predator cue. These reactions can differ considerably from that to actual, free-moving predators [16]–[18]. Real predators are also using the habitat non-randomly and their patch choice may depend on many factors, including temperature and the local prey density.

Mitchell and Angilletta [2] recently published a model (the thermal game model) in which prey must simultaneously gain energy and avoid predators and in which predators can respond to the behavior of their prey. The model provides an interesting prediction of how predator lethality should affect a prey’s choice of thermal patches. According to the model, prey individuals should select thermally optimal habitat patches when predation risk is small, but should increasingly divide their time among patches of different thermal quality as predation risk rises. The predators are expected to reside in the thermally optimal patches for prey more often under all situations. Only if their lethality is very high, they are anticipated to devote a limited amount of their time budget to suboptimal patches. Empirical tests of these predictions of the thermal game model are lacking.

Here, we used a study system featuring dragonfly nymphs as predators and newt larvae as prey to evaluate the thermal game model. We tested the model’s predictions in laboratory and semi-natural settings. In the lab, we exposed individual newt larva to the unrestrained predatory nymph (satiated or hungry) in a stable vertical distribution of warm and cold patches. In the outdoor experiments, we examined the robustness of our laboratory results under more realistic conditions: a group of newt larvae in the presence of free-moving predator was exposed to natural variation in light and temperature. In both settings, we provided control groups with the sole presence of prey and predator. We obtained the proportions of time spent by prey and predator in warm habitat patches in both set-ups and compared them to the model’s predictions.

## Materials and Methods

### Ethics Statement

All experimental procedures were approved by the Expert Committee for Animal Conservation of the Institute of Vertebrate Biology AS CR (research protocol no. 113 / 2009). The Agency for Nature Conservation and Landscape Protection of the Czech Republic issued permission to capture the newts (1154/ZV/2008).

### Study Species

The alpine newt, *Ichthyosaura* (formerly *Triturus*) *alpestris*, is a medium-sized newt distributed over much of central and southern mountainous Europe. Depending on the temperature, larval development lasts between two and four months. When given the opportunity, larvae thermoregulate by selecting locations with favorable temperatures [19]. This behavior is especially pronounced near metamorphosis, when larvae regulate their body temperatures more precisely and at higher levels [20]. Previous behavioral experiments have demonstrated that newt larvae actively avoid dragonfly nymphs (R. Smolinský and L. Gvoždík, unpublished manuscript).

The southern hawker (*Aeshna cyanea*) is a common dragonfly throughout Europe. In our study population, the larval period lasts two years. The nymphs are visually-oriented predators that prey on a variety of aquatic invertebrates and amphibian larvae [21]. Dragonfly larvae may switch between active and ‘sit and wait’ foraging modes depending on circumstances [22]. *Aeshna cyanea* larvae have a wide preferred body temperature range [19], and can be considered thermal generalists. In this respect, our study system complies with the assumption of the thermal game model developed by Mitchell and Angilletta [2], which states that the predator is not selecting microhabitats on the basis of operative temperatures, but solely on the presence of prey.

### Maintenance

Newt larvae were obtained by breeding adult newts collected near Jihlava, Czech Republic. Larvae were reared in ten fiberglass tanks (90×63×47 cm high) filled with 200 l of water. Tanks were provided with aquatic weeds, plankton, and birch forest soil two weeks before introduction of the newts (see [23] for details) and were placed outdoors in partial shade. Preliminary measurements showed that under these conditions, tank water temperatures approached those of water in natural newt habitats [24]. Tank water temperatures were recorded at hourly intervals using dataloggers (resolution 0.5°C; DS1921G-F5, Maxim Integrated Products, Sunnyvale, CA, USA), placed at the bottom of the tanks and near the water surface. Light intensity (lx; wavelength response: 150–1200 nm) was measured using dataloggers (UA-002-08, Onset Computer, Bourne, MA, USA) located on the water surface, in exposed and shaded areas. When a female had deposited 200 eggs, the adult newts were removed from the tank and released at the site of capture.

As the presence of predator chemical cues affects the phenotypes of newt larvae [25], [26], each tank was provided with a dragonfly nymph confined in a floating tube with meshed openings. The nymphs were fed with equal amounts of newt larvae at three-day intervals. The newt larvae were fed regularly by adding equal amounts of zooplankton to each tank. Larval development was monitored weekly. For the experiments, we used larvae at their climax stage (i.e., all digits fully developed; total body length: 36±4 mm). We sampled larvae randomly from the whole stock (*n* ≈ 2000) for behavioral experiments because in a previous study [23] we found minor effects of female and block identity on larval phenotypes kept under similar conditions.

Groups of one-year-old dragonfly nymphs (*n* = 80; total length: 32±5 mm) were kept in four tanks (20 nymphs per tank) filled with 90 l of well water. Tanks were placed outdoors under the same temperature and light conditions as those experienced by newt larvae two weeks prior to the start of the experiments. We used one year-old nymphs as younger individuals are not dangerous to newt larvae at the climax stage. A previous study confirmed that a period of two weeks is sufficient for acclimatization [19]. Nymphs were fed with newt larvae at three day intervals. Just prior to experimentation, we manipulated the ‘lethality’ of the nymphs by applying two feeding regimes: half of the larvae were starved for seven days; the others were placed into individual floating tubes (see above) and fed *ad libitum* for 24 hrs before trials. This allowed us to observe the newt larvae’s behavior under three levels of predation risk: ‘no risk’ (predator absent), ‘low risk’ (fully fed predator), and ‘high risk’ (hungry predator). We used each dragonfly nymph and newt larva in one trial only.

### Laboratory Experiment

Laboratory observations were conducted in an environmental room kept at 12°C and involved eight glass aquaria (60×10×40 cm high) filled to 30 cm with thermally stratified water [27]. In each aquarium, a heater (50 W, Eheim/Jäger, Wüstenrot, Germany), located 5 cm below the water surface, created a vertical temperature gradient. The heater was placed in a recess separated from the rest of aquarium by a mesh so as not to affect the behavior of the test animals. Temperatures in the aquaria ranged from 20–25°C at the top of the water column to 13–14°C at the bottom. The higher temperatures correspond to the preferred body temperature range of newt larvae at climax stage (21–26°C; [20]). The aquaria were equipped with profiled bottoms (10 cm flat, 40 cm inclined, and 10 cm flat) that allowed the newt larvae to bottom-walk to patches with different temperatures. In one extra aquarium that was not used for behavioral observations, a series of thermistor probes connected to dataloggers (H08-002-02, Onset Computer, Bourne, MA, USA) recorded hourly water temperatures at 5 cm intervals in the water column. In the other aquaria, the temperature of the warm patch (i.e. the higher flat area) was checked regularly to ensure that thermal conditions were similar throughout the experiments.

Twelve hours prior to the observations, one randomly selected newt larva was placed separately in each of the eight test aquaria. At 0800 am, one dragonfly nymph (either satiated or hungry) was added to four of the aquaria. We recorded the prey’s and predator’s positions in the water column (5 cm intervals) hourly between 0900 h and 1700 h. Pilot tests ensured that the presence of human observers did not affect behavior in either study species. In an additional series of experiments, we compared the behavior of dragonfly nymphs (satiated versus hungry) in aquaria without newt larvae (predator-only treatment).

### Semi-natural Experiment

The outdoor experiment involved 19 fiberglass tanks (90×63×47 cm high) that were half-buried in a sandy soil. The containers had profiled bottoms similar to that of the laboratory aquaria (15 cm flat, 60 cm inclined, 15 cm flat). The tanks were placed in two rows from east to west, in such a way that each experimental tank had its control counterpart, with similar light and temperature conditions. Two calibrated dataloggers (DS1921G-F5, Maxim Integrated Products) recorded water temperatures near the water surface (25±0.2°C, *n*_readings_ *= *675) and at the bottom (18±0.1°C). Groups of randomly chosen newt larvae (*n* = 10) were placed into each tank 12 hrs before the beginning of a trial. One randomly chosen dragonfly nymph was placed in ten of the tanks one hour before a trial. We slowly walked along the set-up and noted the proportion of newt larvae occupying the warm patch (i.e. the higher flat area).

### Statistical Analyses

We used generalized linear mixed-effect models (GLMM) to test predictions of the thermal game model. Laboratory data were analyzed as logit-transformed proportions of time that the newt larvae or dragonfly nymphs spent in the warm patch. For the dragonfly nymphs, the full model consisted of one fixed (treatment: satiated versus hungry) and one random (experimental aquarium) factor. The treatment contrasts for newt larvae were specified to enable two orthogonal comparisons; predator absence vs. predator presence and satiated (low lethality) predator vs. hungry (high lethality) predator.

Since outdoor thermal conditions varied considerably during and among days, we added ‘water temperature in warm patch’ as a covariate to the model. We used the larval group identity nested within the experimental tank as a random grouping factor. The dependent variable was the hourly proportions of animals observed in the warm patch. The dragonfly nymphs data were analyzed as the presence/absence of the animal in the warm patch. In case of significant over-dispersion, the quasi-binomial alternative of GLMM was applied. The minimum adequate model selection was realized using the likelihood ratio approach [28]. All analyses were performed in the R programming environment (version 2.15.0; [29]) using the ‘lme4’ [30] and ‘MASS’ [31] libraries. Data used in final analyses are submitted to Dryad and located at doi: 10.5061/dryad.fj168.

## Results

### Laboratory Experiment

**Figure 1:**
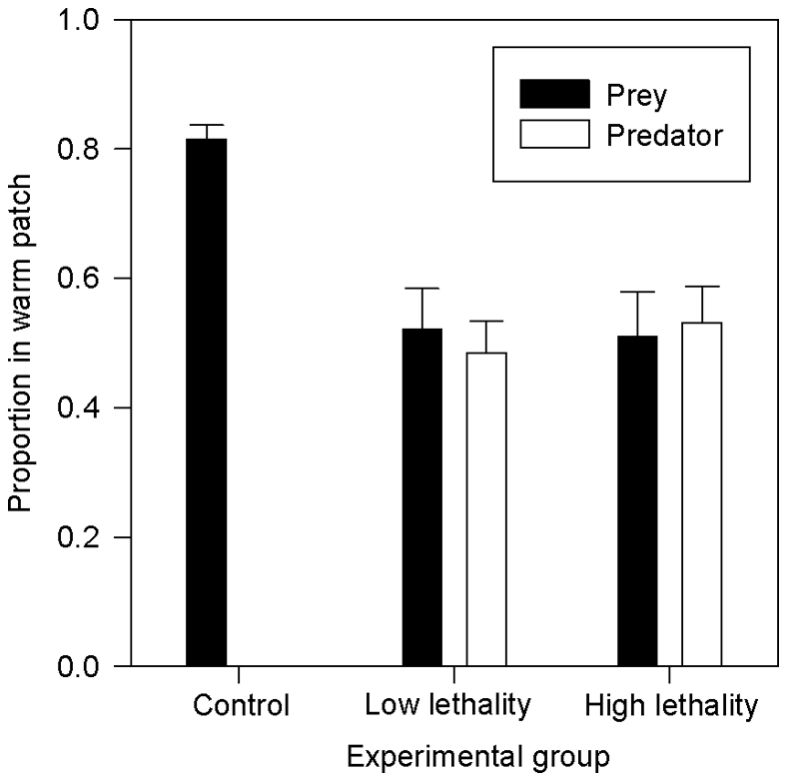
Thermal patch use in laboratory settings. Proportion of time (mean±SE) that prey (newt larvae) spent in the warm patch under the absence (control) and presence of a predator (dragonfly nymph) with two lethality/satiation levels (i.e. hungry and fully fed) in constant laboratory conditions. See fig. 2. for predator control (i.e. prey absent).

**Figure 2:**
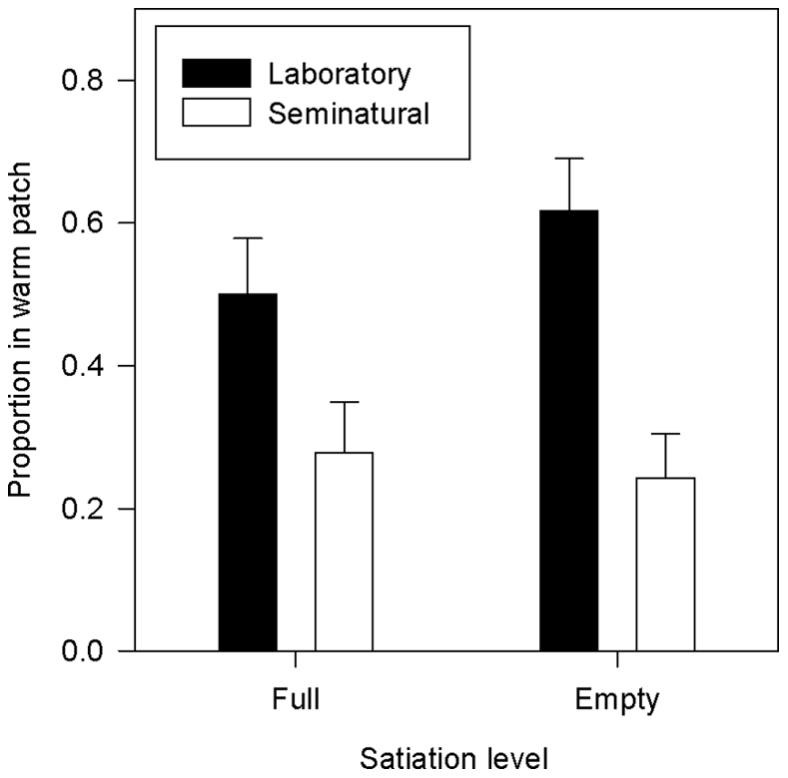
Thermal patch use in sole predator. Proportion of time that fully fed (low lethality) and hungry (high lethality) dragonfly nymph spent in a warm patch in laboratory and semi-natural settings under the absence of prey.

The newt larvae used the warm patch less often when sharing the aquarium with a dragonfly nymph than in solitude (*t*_86_ = 4.59, *P*<0.001; Fig. 1). However, the predator’s satiation level (i.e., its lethality) did not influence the time newts spent in the warm patch (*t*_86_ = 0.13, *P* = 0.89). Contrary to model predictions, the satiation level of dragonfly nymphs did not affect their patch choice. Regardless of whether prey was present (*t*_39_ = 0.95, *P* = 0.35) or absent (*t*_22_ = 1.46, *P* = 0.16), the nymphs spent similar amounts of time in the warm patch when satiated or hungry (Fig. 2). The proportion of time the predator spent in the warm patch was not correlated to the time spent there by the prey (satiated nymphs: *r*_Spearman_ = 0.25, *P* = 0.25; hungry nymphs: *r*_Spearman_ = −0.25, *P* = 0.25).

### Semi-natural Experiment

**Figure 3:**
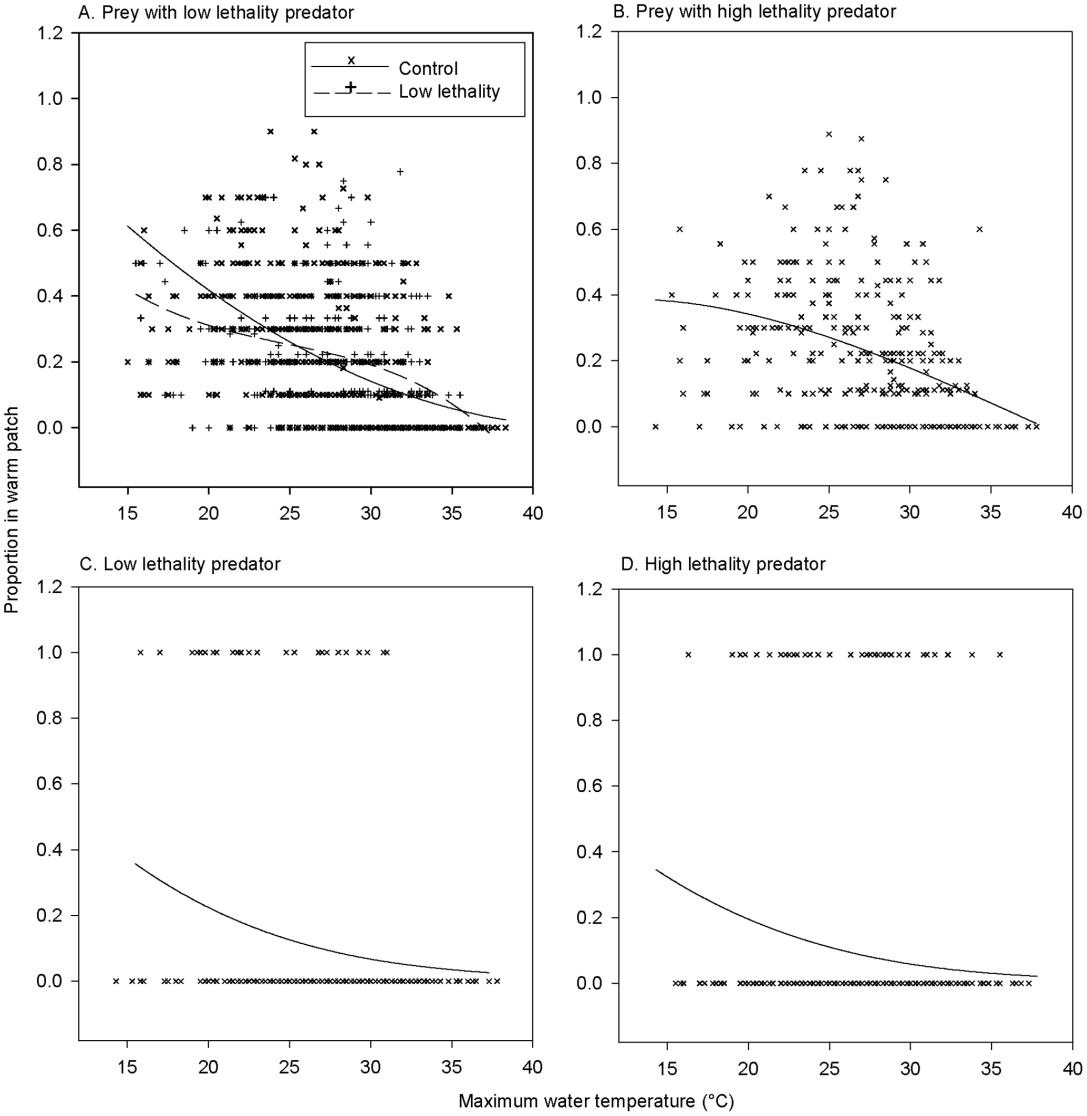
Thermal patch use in seminatural settings. The relationship between maximum water temperature and the proportion of prey (newt larvae) and predator (dragonfly nymph) in the warm patch, under semi-natural conditions. In the experimental situations (but not in the control situation), prey and predator were placed in the aquaria together, without any barrier between them. Lines are fits of generalized linear mixed-effect model.

In the semi-natural experiments of 2011, acute water temperature was a strong predictor of the presence/absence in the warm patch for both newt larvae (*t*_1193_ = 6.19, *P*<0.001) and dragonfly nymphs (*t*_628_ = 4.81, *P*<0.001). This relationship varied among treatments in newt larvae (temperature×treatment: *t*_1193_ = 4.33, *P*<0.001). In water temperatures below 25°C, individuals from the prey-only treatment occupied the warm patch more frequently than individuals in the presence of predators (Fig. 3A, 3B). In 2012, weather conditions were much less variable and water temperature had no demonstrable influence on the number of nymphs seen occupying the warm patch (χ^2^ = 0.97, df = 1, *P* = 0.32). As in the laboratory, the proportion of dragonfly nymphs residing in the warm patch was independent of their hunger status (with newt prey: χ^2^ = 0.02, df = 1, *P* = 0.89; without prey: *t*_17_ = 0.37, *P* = 0.72; Fig. 2, 3C, 3D). Starving nymphs killed more larvae than fully satiated individuals (χ^2^ = 4.55, df = 1, *P* = 0.03; low lethality = 0.042±0.01; high lethality = 0.083±0.01).

## Discussion

In previous experimental studies of the interaction between predation and thermoregulation, predators have typically been ascribed a largely passive role. Also, experiments have generally been conducted in overly simplistic laboratory settings, and it is unclear whether the emanating results can be extrapolated to more natural conditions. Here, we aimed to conduct more realistic tests by assessing the prey species’ thermal decisions in the presence of unrestrained predators, and by running a parallel experiment in semi-natural environments.

The thermal game model [2] predicts that whereas prey should select habitat patches depending on local thermal conditions and predation risk, predators should choose habitat patches solely on the basis of prey spatial behavior. In our laboratory experiments, *Ichthyosaura alpestris* newt larvae (the prey species) followed the model’s predictions, curtailing their use of the warm patch in the presence of predators. The same interaction between thermoregulation and predator avoidance behavior was observed in the experiment in semi-natural conditions. In contrast, the thermal game model failed to predict the behavior of the *Aeshna* dragonfly nymphs (the predator species) in both the laboratory and semi-natural experiments: the nymphs did not spend more time in warm patch than prey.

We found it encouraging that adding system complexity (semi-natural versus laboratory set-up) did not fundamentally change the outcome of our experiments. This seems to suggest, on the one hand, that behavioral observations in relatively simple set-ups may provide ecologically relevant information on fairly complex decision-making in these animals. On the other hand, the fact that our empirical test provides only partial support for the thermal game model requires further theoretical work on the issue.

The restrained use of the thermally favorable patches by prey not only supports predictions of the thermal game model. It also corroborates the more general spatial distribution model [32], [18], if temperature is considered a resource [33]. As the newts’ body temperatures probably depend on the amount of time spent in the warm patch, our results are also in line with earlier empirical studies on the thermoregulatory costs of predator avoidance, where prey maintain lower body temperatures in the presence of predator cues [34], [35], [13].

In our study system, the prey’s strategy of limiting the time spent in warm patches when predation risk is high seems sensible, because raising temperatures indeed raise the larvae’s susceptibility to predation [23]. As the dragonfly nymphs spent less time in the warm patch than newt larvae, it seems that the prey’s patch choice is not so much shaped by the predator’s actual position (as previous studies suggest), but rather by the presence of a predator in the experimental tank as a whole. If this holds also in natural communities, the thermal decisions of newt larvae are influenced by the overall occurrence of predators, rather than by the variation in predation costs among patches.

According to the thermal game model, the effect that predators have on their prey’s thermal habitat use will modify selection pressures on the prey’s thermal physiology traits [2]. Specifically, the presence of predators is predicted to favor the evolution of wide thermal performance curves (thermal generalists) in prey species. This may help us to understand why physiological performance of prey species at low temperatures is high compared to that of predator species [36]. In our study population, the occurrence of dragonfly nymphs varies among pools (J. Dvořák, R. Smolinský and L. Gvoždík, unpublished observations; see also [25]), which may increase among-generation relative to within-generation variation in body temperatures of newt larvae. These conditions should favor thermally-induced developmental plasticity (developmental acclimation) over fixed phenotypes [37]. Indeed, a plastic response to developmental temperatures has been reported for several traits in Alpine newt larvae [38], [39], [19], and many other ectotherms [40], [33]. How biotic interactions shape the evolution of both fixed and plastic thermal performance curves in thermally heterogeneous environment poses an interesting research plan for further theoretical studies.

Why do the *Aeshna* nymphs fail to comply with the thermal game model’s predictions? Of many possible explanations [18], we highlight three. First, the *Aeshna* nymphs may have based their patch choice on thermal considerations, rather than on prey spatial behavior (as assumed by the model). Although all water temperatures in the laboratory set-up fall within the nymphs’ preferred temperature range [19], the temperature of the warm patch was closest to the upper bound of this range. Thus the warm patch may have functioned as a ‘spatial anchor’ in Sih’s [18] terminology. Future thermal games models should consider the possibility of such anchors in both predator and prey species.

Second, in their model, Mitchell and Angilletta [2] assumed that predator fitness depends exclusively on the number of captured prey and the effectiveness of assimilation rates, whereas prey fitness is determined by energy gain and predation risk. However, if predators are not starving to death [18], their relationship between patch choice and fitness should be much weaker than in prey due to the life-dinner principle [41]. If predators less closely follow the model, then prey should show a stronger preference for the warm patches.

Third, the predator’s ability to select patches of high prey density will depend on its ability to recognize the (thermal) value that distinctive patches represent for their prey. However, the preferred body temperatures of newts (and hence, the value of particular patches) vary ontogenetically and seasonally [20], [19]. This may seriously complicate the predator’s task of recognizing patches that are thermally favorable to prey. That is, the overall variation in preferred body temperatures may indirectly contribute to predator avoidance in newts.

Although the results from our two series of observations were consistent, both newt larvae and dragonfly nymphs clearly spent less time in the warm patches outdoors than in the laboratory. A main difference between both set-ups was that newt larvae were kept in groups in the outdoor tanks, so the lower occupancy of warm patches in these tanks could be attributed to intra-specific competition among newts (cf. [42]–[44]). However, this explanation does not hold for the dragonfly nymphs. Alternatively, dragonfly nymphs and newts may prefer to curtail activity near the water surface to prevent attracting the attention of aerial predators [45].

In summary, our study revealed an asymmetric behavioral response in a predator-prey system: the presence of a predator affected the prey’s thermoregulatory behavior, but the predator did not change its choice of habitat in response to the spatial behavior of prey. This uneven response is visible even under semi-natural conditions. Our findings only partially corroborate predictions of the thermal game model. We advocate the use of unrestrained predators in future empirical tests of the model and emphasize the need to consider the thermal requirements of prey and predator species and the thermal heterogeneity of environment in studies on predator-prey space use. Thermal game models seem a promising tool for exploring how biotic interactions may influence an ectotherm’s response to sustained climate change [46]–[48].
